# Recent research progress on metal ions and metal-based nanomaterials in tumor therapy

**DOI:** 10.3389/fbioe.2025.1550089

**Published:** 2025-02-07

**Authors:** Yongcheng Xu, Aikebaier Reheman, Wenhua Feng

**Affiliations:** ^1^ The Second School of Clinical Medicine, Shenyang Medical College, Shenyang, China; ^2^ Fujian Key Laboratory of Toxicant and Drug Toxicology, Medical College, Ningde Normal University, Ningde, Fujian, China; ^3^ Department of Human Anatomy, School of Basic Medicine, Shenyang Medical College, Shenyang, China; ^4^ Liaoning Province Key Laboratory for Phenomics of Human Ethnic Specificity and Critical Illness, Shenyang, China; ^5^ Shenyang Key Laboratory for Phenomics, Shenyang Medical College, Shenyang, China

**Keywords:** metal-based nanomaterials, tumor therapy, ferroptosis, calcium overload, cuproptosis, immunotherapy

## Abstract

Tumors, as a disease that seriously threatens human health, have always been a major challenge in the field of medicine. Currently, the main methods of tumor treatment include surgery, radiotherapy, chemotherapy, etc., but these traditional treatment methods often have certain limitations. In addition, tumor recurrence and metastasis are also difficult problems faced in clinical treatment. In this context, the importance of metal-based nanomaterials in tumor therapy is increasingly highlighted. Metal-based nanomaterials possess unique physical, chemical, and biological properties, providing new ideas and methods for tumor treatment. Metal-based nanomaterials can achieve targeted therapy for tumors through various mechanisms, reducing damage to normal tissues; they can also serve as drug carriers, improving the stability and bioavailability of drugs; at the same time, some metal-based nanomaterials also have photothermal, photodynamic, and other characteristics, which can be used for phototherapy of tumors. This review examines the latest advances in the application of metal-based nanomaterials in tumor therapy within past 5 years, and presents prospective insights into the future applications.

## 1 Introduction

There are various essential metal ions in the human body that participate in many important life activities, such as signal pathway activation, enzyme catalysis, and protein composition (Lv et al., 2023). In normal cells, theprecise regulation of ion homeostasis is crucial for cell survival, metabolism, and immunity (Liu et al., 2024). The abnormal distribution or accumulation of certain metal ions can activate cell toxicity-related biochemical reactions and induce cell death. Based on this principle, a new treatment strategy has emerged, namely, metal ion-mediated tumor treatment, which inhibits tumor growth by directly or indirectly regulating the concentration of metal ions within cells (Feng et al., 2023; Sun et al., 2024). In addition, metal ions also play a key role in tumor immune regulation, and the concept of “cancer metal immunotherapy” has been proposed (Wang et al., 2023c). Therefore, an increasing number of compounds with the ability to regulate metal ions (such as curcumin, deferoxamine, disulfiram) have been developed for tumor treatment research (Wu et al., 2020). However, their clinical transformation is hindered by limited specific recognition ability and inadequate ion concentration changes. In recent years, nanomaterials have made significant progress and development in drug delivery, diagnosis and imaging, treatment, vaccines, and other fields (Wang G. et al., 2023). Various metal ion-regulated nanomaterials have also made great progress in tumor treatment research. They not only optimize the metal ion-based antitumor treatment system but also provide the possibility for the combination of metal ion treatment with other treatment strategies. These characteristics make metal-based nanomaterials show great potential for tumar treatment.

This article will comprehensively summarize the diverse types, intricate mechanisms, and the latest research advancements in the field of metal-based antitumor nanomaterials and offer insights into the anticipated directions of development.

## 2 Metal ions and tumors

### 2.1 The role of iron in antitumor therapy

#### 2.1.1 Physicochemical properties, physiological functions of iron and ferroptosis

Iron (symbol: Fe) is the 26th metal element in the periodic table, with valences of Fe^2+^, Fe^3+^, and Fe^6+^, among which Fe^2+^ and Fe^3+^ are the most common and can convert into each other in the human body. Iron is an important essential trace element in the human body, mainly existing in two forms: heme iron and non-heme iron. Heme iron is an important component of hemoglobin in red blood cells and myoglobin in muscle, responsible for oxygen transport in the blood and oxygen storage in the muscle, respectively (Sawicki et al., 2023). Non-heme iron combines with iron-binding proteins, such as ferritin and hemosiderin. In addition to participating in hemoglobin synthesis and oxygen transport, iron also acts as a cofactor for various enzymes involved in many important cellular processes, such as DNA synthesis and repair, electron transfer, and respiration (Powell et al., 2023). The maintenance of iron homeostasis is crucial for the normal life activities of the body.

**FIGURE 1:**
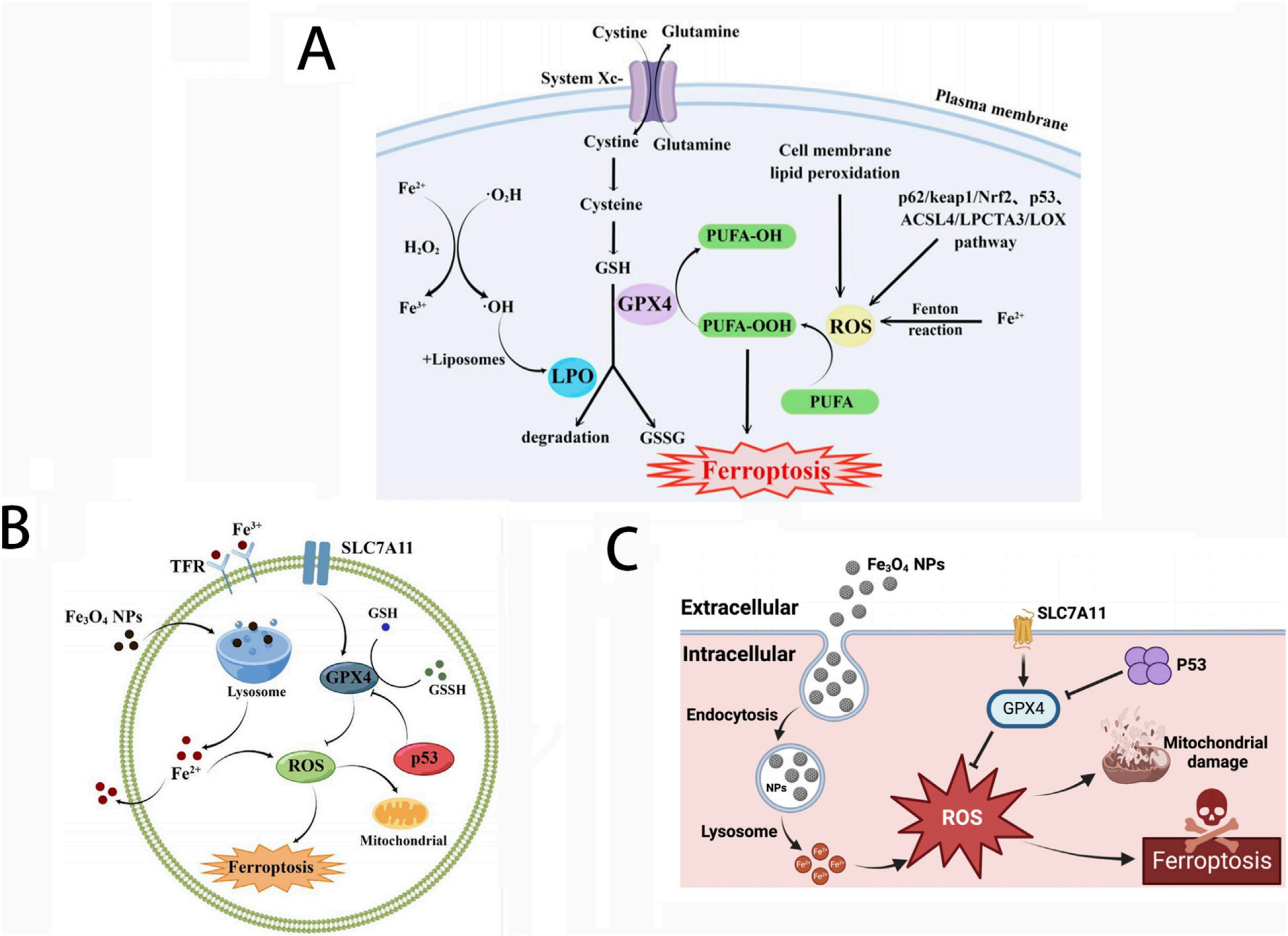
**(A)** Major metabolic pathways of ferroptosis; **(B)** Effect of Fe_3_O_4_-NPs on cell ferroptosis; **(C)** Mechanism of Fe_3_O_4_-NP-induced ferroptosis through upregulation of p53. Reproduced with permission from ref (Wang et al., 2023d); CC BY 4.0. Copyright ^©^ 2023 by the authors.

Inside cells, iron homeostasis is precisely regulated at the transcriptional and translational levels by iron regulatory proteins (IRPs) and iron response elements (IREs) to control iron uptake, storage, and efflux, as well as the management and distribution of iron within cells (Billesbolle et al., 2020). Fe^2+^ is the main active form of iron, participating as a structural or catalytic cofactor in redox reactions. When iron levels surge beyond normal, an abundance of Fe^2+^ can trigger oxidative stress and disrupt the equilibrium of the antioxidant defense system. Excess free radicals, generated through redox reactions, attack the polyunsaturated fatty acids within the cell membrane, thereby triggering a chain reaction of lipid peroxidation, which in turn leads to cell death, a phenomenon designated as ferroptosis. Ferroptosis is a programmed iron ion-dependent cell death mechanism that plays a role in various physiological and pathological processes (Mou et al., 2019; Nguyen et al., 2023). Ferroptosis is mainly caused by the excessive accumulation of intracellular iron ion-dependent reactive oxygen species (ROS) and the weakened elimination of glutathione peroxidase 4 (GPX4), which causes the homeostatic imbalance of ROS generation and degradation. When the cell’s own antioxidant capacity is insufficient to remove excessively accumulated lipid ROS, it causes ferroptosis. Iron accumulation and subsequent lipid peroxidation play an important role in mediating the occurrence of ferroptosis. Thus, the various molecules and signals involved in iron metabolism and lipid peroxidation are all critical for regulating iron death. The changes in cell metabolic pathways caused by ferroptosis mainly include inhibited GSH synthesis and LPO accumulates, iron metabolism and ROS metabolic pathway (as shown in Figure 1).

#### 2.1.2 Antitumor mechanisms based on ferroptosis

Iron metabolism disorders are closely related to the occurrence and development of tumors. Since iron is an essential element for cell proliferation and metabolism, the rapid proliferation of tumor cells shows an abnormal demand for iron, so tumor cells promote the accumulation of iron in cells through iron metabolism reprogramming (Yang et al., 2023). Therefore, the iron content in tumor cells is higher than in normal cells. Due to the influence of the tumor microenvironment, tumor cells are more susceptible to ferroptosis inducers than normal cells, creating a fundamental prerequisite to undergo anti-tumor treatment utilizing ferroptosis (Lei et al., 2022). However, the vast majority of iron within tumor cells predominantly exists in the form of non-cytotoxic ferritin. Therefore, targeting ferritin to facilitate the liberation of iron ions or directly modulating the intracellular iron ion concentration are two feasible ferroptosis-based anti-tumor treatment strategies.

In addition, since the concentration of hydrogen peroxide (H_2_O_2_) in tumor cells is relatively higher than in normal cells, excessive iron ions will undergo the Fenton reaction with H_2_O_2_ (Huang L. et al., 2023). In the process of this reaction, the interaction between Fe^2+^ and H_2_O_2_ leads to the production of hydroxyl radicals (·OH), a highly biologically toxic ingredient capable of damaging lipids, proteins, DNA, and other vital cellular components, ultimately leading to the death of tumor cells. Therefore, increasing the concentration of free Fe^2+^ and H_2_O_2_ within tumor cells at the same time can mediate cell death through the Fenton reaction, which may become one effective anti-tumor strategy based on iron ion.

#### 2.1.3 Iron ion-based anti-tumor nanomedicines

With the in-depth study of ferroptosis and the development of nanotechnology, nanomaterials have been proven to induce ferroptosis more effectively than biological drugs. First, it can break the iron homeostasis both through *ex vivo* delivery mechanisms and endogenous iron utilization pathways, enhancing the elevation of intracellular free iron levels. Currently, various nanodrugs containing Fe^2+^/Fe^3+^ or capable of delivering Fe^2+^/Fe^3+^ have been developed for anti-tumor treatment, such as polydopamine (PDA) and amorphous CaCO_3_ nanoparticles, etc (Han et al., 2022). Iron-bearing nanoparticles can not only release Fe^2+^ in acidic lysosomes but also deliver antitumor medications, such as a chemotherapeutic drug cisplatin, achieving a synergistic antitumor effect (Cheng et al., 2021). Biocompatible iron nanoparticles, Fe_3_O_4_-NPs, not only promote the production of reactive oxygen species (ROS) but also participate in iron metabolism, leading disruption of intracellular iron balance and induction of ferroptosis. In addition, Fe_3_O_4_-NPs combined with other technologies, such as photodynamic therapy (PDT), thermal stress, and sonodynamic therapy (SDT), can further induce cellular ferroptosis, thereby enhancing the antitumor effects (Wang et al., 2023d; Yao et al., 2021).

Another method to increase the level of free Fe^2+^ within cells is to target iron-related proteins, among which ferritin degradation is considered an effective approach. A ferritin-hijacking nanoparticle (Ce6-PEG-HKN_15_) is fabricated, by conjugating the ferritin-homing peptide HKN15 with the photosensitizer chlorin e6 (Ce6) for endogenous ferroptosis without introducing Fenton-reactive metals (Zhu et al., 2022). Since the HKN15 peptide can target ferritin, the photosensitizer chlorin e6 (Ce6) can specifically aggregate around ferritin. Under laser irradiation, the activated Ce6 in these nanoparticles synergizes with the generated ROS to effectively destroy ferritin and release Fe^2+^. In turn, the released iron partially interacts with intracellular excess H_2_O_2_ to produce O_2_, thereby enhancing photodynamic therapy and further amplifying oxidative stress. Xiong et al. prepared a nano-activator (DAR) which was assembled by doxorubicin (DOX), tannic-acid (TA) and IR820 as a photosensitizer to make full use of endogenous iron stored in endo-lysosome, realizing ferroptosis and its related oxidative stress through artificially intracellular positive feedback loop, providing an innovative solution for the development of antitumor treatment based on ferroptosis-immunotherapy (Xiong et al., 2021).

### 2.2 The role of calcium in antitumor therapy

#### 2.2.1 Physicochemical properties and physiological functions of calcium

Calcium (symbol: Ca) is a chemical element with an atomic number of 20, and its ionic form is Ca^2+^. Calcium is the most abundant metal element in the human body and is crucial for the formation of bones and teeth. In the body, calcium exists in three main forms: free calcium, complex calcium, and protein-bound calcium, which can convert into each other. Among them, free calcium is the only physiologically active form. As an indispensable second messenger in cells, calcium ions (Ca^2+^) participate in the regulation of almost all physiological processes by activating specific target proteins. Due to the importance of Ca^2+^, its concentration is strictly controlled (Berridge et al., 2000; Carafoli, 2002; Liu et al., 2020; Marchi et al., 2020; Monteith et al., 2017). The concentration of free calcium within cells is only 100 nM, much lower than the extracellular calcium concentration (Bagur and Hajnoczky, 2017). Fluctuations in Ca^2+^ concentration will affect normal calcium signal transmission and thus affect cellular physiological functions. The homeostasis of intracellular Ca^2+^ mainly relies on the orderly cooperation of various Ca^2+^ channels in the cell membrane and organelles (such as the endoplasmic reticulum, mitochondria, and lysosomes) (Berridge, 2016; Clapham, 2007; Huang et al., 2022). When this homeostatic mechanism is disrupted, excessive intracellular calcium ion concentration may lead to calcium overload, resulting in cell death (Hof et al., 2019).

#### 2.2.2 Antitumor mechanism based on calcium overload

**FIGURE 2:**
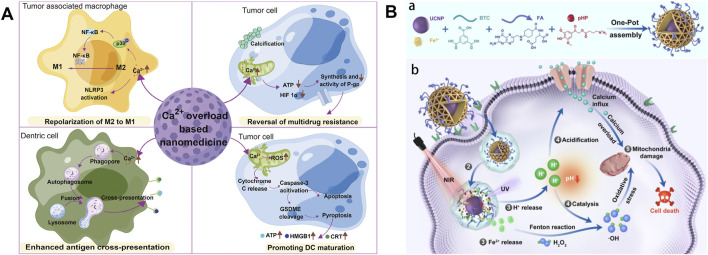
**(A)** Schematic diagram of antitumor mechanisms of Ca^2+^ overload-based nanomedicines. Reproduced with permission from ref (Xu et al., 2024). **(B)** Schematic Illustration of the construction of FMUP nanoagent and the underlying anticancer mechanism. **(a)** Core-shell type FMUP nanoagents with UCNP as the core and photoacid (pHP) encapsulated in the cavity of FA-doped MOFs shell were constructed by one-pot self-assembly. The FMUP nanoagent was synthesized by coordination of carboxyl groups on BTC and FA with Fe^3+^. The UCNP as the core was located in the shell and simultaneously the pHP was loaded in the pore of the nanoagent. **(b)** Corresponding anticancer mechanism of FMUP nanoagent. ① Active internalization of FMUP in tumor cells helped by FA functionalization. ② Lysosome escape of FMUP induced by the increase of osmotic pressure after NIR light irradiation. ③ Fe^2+^ and H^+^ release from FMUP upon NIR light irradiation. ④ Photoacidification of intracellular microenvironment induced calcium influx and therefore calcium overload in the mitochondria and simultaneously generated a key acidic environment for efficient Fenton reactions. The release of Fe^2+^ and photoacidification synergistically reinforced Fenton reactions and therefore produced a large number of ·OH within the close proximity of mitochondria. ⑤ As a result, the calcium overloaded and plentiful ·OH enabled dual damage to mitochondria and further induced cell death. Reproduced with permission from ref (Bao et al., 2021); CC BY 4.0. Copyright ^©^ 2023 by the authors.

Ca^2+^ is the most abundant metal element and the second messenger in the human body, regulating specific biological functions that involve all aspects of cell life, and closely participate in the formation, proliferation, and migration of tumor cells (Bagur and Hajnoczky, 2017; Wu Y. et al., 2024). The expression of Ca^2+^ signal pathway-related proteins in tumor cells is different from that in normal cells, and the Ca^2+^ signal pathways are related to the pathogenesis of specific tumors such as breast cancer, colon cancer, lung cancer, liver cancer, etc (Jing et al., 2016; Wang et al., 2019; Zhang et al., 2015). Compared to normal cells, tumor cells such as breast cancer, prostate cancer, and melanoma exhibit reduced Ca^2+^ influx mediated by stromal interaction molecules to avoid cell death caused by intracellular Ca^2+^ overload. This mechanism promotes the proliferation of tumor cells (Dubois et al., 2014; Faouzi et al., 2013; Sun et al., 2014). Therefore, tumor cell death caused by calcium overload can be achieved by correcting the abnormal Ca^2+^ signaling pathway or introducing exogenous Ca^2+^ into the cytoplasm via calcium ion carriers (Cao et al., 2024). When a large amount of Ca^2+^ enters the mitochondria, it can inhibit the synthesis and biological activity of drug-resistant proteins, promote the uptake and retention of cells for anticancer drugs. In addition, mitochondrial Ca^2+^ overload can also increase the level of intracellular ROS, causing the release of cytochrome C (Cyt C), thereby activating cysteine protease 3 (caspase-3) and GSDME protein, jointly promoting the occurrence of cell pyroptosis (as shown in Figure 2) (Bao et al., 2021; Xu et al., 2024; Yu et al., 2021). In summary, nanodrugs that can directly introduce Ca2^+^ into cells or organelles can provide new ideas for tumor treatment.

#### 2.2.3 Calcium-based antitumor nanodrugs

With the continuous development of nanotechnology, the functions of nanomaterials are constantly being engineered. The emergence of calcium-based nanocomposites has well solved the problem of low efficiency in directly delivering Ca^2+^ to tumor tissues, greatly expanding the application of Ca^2+^ in the field of tumor treatment (Bai et al., 2022). Calcium-based nanocomposites used in tumor treatment include calcium phosphate (CaP), calcium carbonate (CaCO_3_), calcium peroxide (CaO_2_), etc. (Li et al., 2021; Xu et al., 2018; Zhao et al., 2021; Zheng et al., 2021), which have excellent pH response capabilities and are often used in the design of pH-dependent nanodrug delivery platforms (An et al., 2020). In addition, calcium-based nanocomposites can also target tumor tissues or cells. After internalization, they can degraded under the action of acidic organelles (such as lysosomes), and release a large amount of free Ca^2+^. This process, when combined with Ca^2+^ influx promoters or Ca^2+^ efflux inhibitors, can further elevate the intracellular Ca^2+^ concentration that consequently induce cell apoptosis due to Ca^2+^ overload (Gao S. et al., 2019). Therefore, calcium-based nanodrugsits are very promising material for antitumor treatment.

### 2.3 The role of manganese in antitumor therapy

#### 2.3.1 Physicochemical properties and physiological functions of manganese

Manganese (element symbol: Mn) is a transition element with an atomic number of 25, having various valence states, including Mn^2+^, Mn^3+^, Mn^4+^, Mn^6+^, and Mn^7+^. The most common form of manganese found in living tissues is Mn^2+^ and Mn^3+^. Manganese plays an important role in various physiological processes, including development, energy metabolism, antioxidant defense, and immune function. Manganese acts as a cofactor for various enzymes in the body, such as arginase, manganese superoxide dismutase (MnSOD), and cyclic guanosine monophosphate-adenosine monophosphate synthase (cGAS), etc. (Liu et al., 2022; Zhu et al., 2019). Among them, the cGAS protein is an important DNA sensor that activates the host immune response, and the cGAS-STING signaling pathway plays a crucial role in innate immune responses (Wang et al., 2018). Therefore, manganese is an important immune activator and the level of manganese in cells also needs precise regulation. Manganese efflux pumps and metal transporters ZIP8, ZIP14, and ZnT10 play a key role in this process (Xin et al., 2017). Due to the chemical properties of strong redox capabilities that similarity to iron, manganese can also produce ·OH through a Fenton-like reaction, increasing oxidative stress and producing cytotoxic effects (Ju et al., 2022).

#### 2.3.2 Antitumor mechanism of manganese

Numerous epidemiological investigations have found a significant positive correlation between low manganese and tumor occurrence (Kim, 2010; Tu et al., 2010). The purpose of antitumor manganese nanodrugs is to increase the concentration of intracellular free Mn^2+^. After nanoparticles are internalized by cells, the release of Mn^2+^ depletes intracellular glutathione (GSH), enabe the sufficient generation of reactive oxygen species (ROS) and effectively kill tumor cells. In addition, Mn^2+^ can also catalyze conversion of cellular H_2_O_2_ to ·OH through a Fenton-like reaction. It also promotes the decomposition of H_2_O_2_ to O_2_ and continuously catalyzes the conversion of O_2_ to cytotoxic ·O^2-^ via oxidase-like activity that enhance the therapeutic effects of radiotherapy and starvation therapy (Zhu et al., 2021).

**FIGURE 3:**
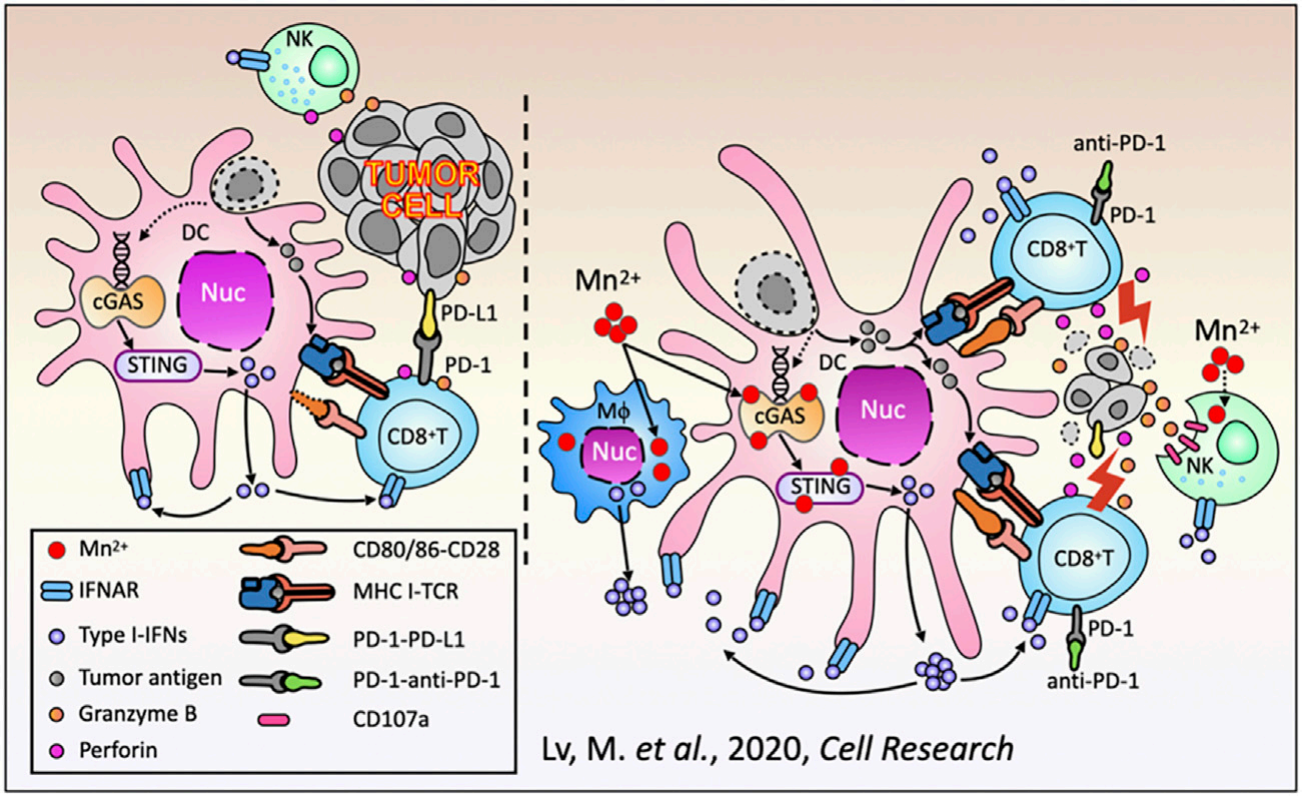
Schematic diagram of the antitumor immune responses of Mn^2+^. Reproduced with permission from ref (Lv et al., 2020); CC BY 4.0. Copyright ^©^ 2020 by the authors.

**FIGURE 4:**
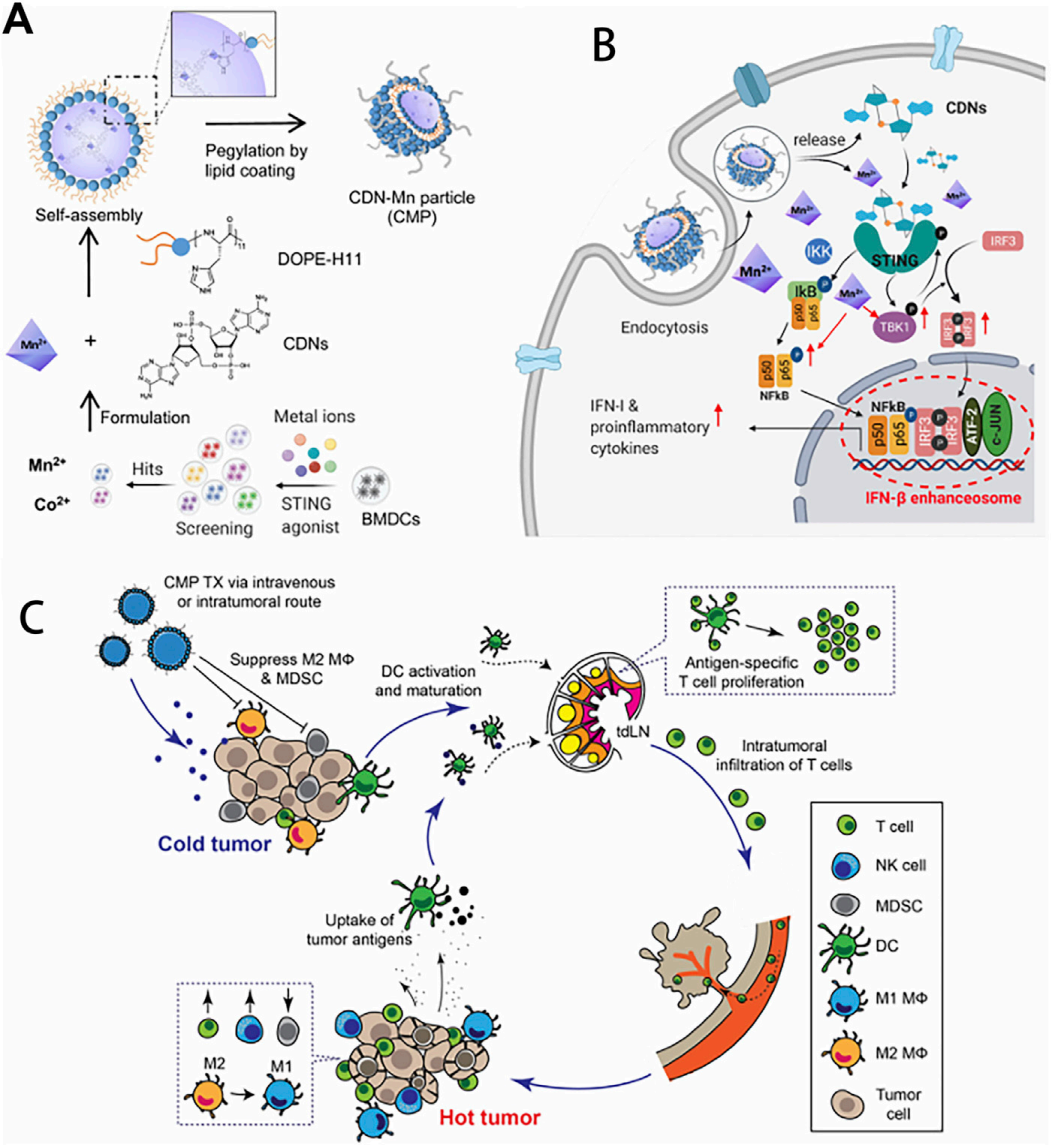
Amplifying STING activation with CDN-Manganese particles (CMP) for cancer metalloimmunotherapy. **(A)** CMP is composed of cyclic di-nucleotides (CDNs), Mn^2+^, DOPE-H11, and a PEG-lipid layer. Mn^2+^ potentiates type-I IFN activities of STING agonists. Mn^2+^ and CDNs self-assemble into coordination polymer. CDN-Mn^2+^ coordination polymer was coated with DOPE-H11 via Mn-histidine coordination to form CDN-Mn@DOPE, followed by PEGylation with PEG-lipid layer, resulting in the formation of CMP. **(B)** CMP boosts STING activation: 1) CMP promotes cellular uptake of CDNs and Mn^2+^; 2) Mn^2+^ augments CDN induced STING activation via STING-independent TBK1 and p65 phosphorylation, STING dependent IRF3 phosphorylation, and assembly of the IFN-β transcriptional enhanceosome. **(C)** CMP exerts potent anti-tumor efficacy after intratumoral (I.T.) or intravenous (I.V.) administration. CMP reverses immunosuppressive tumor microenvironment while activating T-cells, natural killer (NK) cells, and dendritic cells (DCs). Reproduced with permission from ref (Sun et al., 2021); PDM 1.0. Copyright ^©^ 2021 by the authors.

Manganese possesses strong immune activation capabilities. The cGAS-STING signaling pathway plays a crucial role in innate immune responses, where cGAS, as a DNA sensor, can detect double-stranded DNA released into the cytoplasm or extracellularly from damaged tumor cells, triggering an immune response (Wang et al., 2018). As a cofactor for cGAS, Mn^2+^ is a strong activator of the cGAS-STING signaling pathway, which can promote the production of type I interferon (IFN), enhance antigen presentation efficiency, and enhance the differentiation and activation of CD8^+^ T cells (as shown in Figures 3, 4) (Lv et al., 2020; Sun et al., 2021; Wang et al., 2018). After radiotherapy or chemotherapy, the accumulation of Mn^2+^ and the leakage of nuclear DNA can effectively promote the activation of the cGAS-STING signaling pathway, thereby enhancing the antitumor immunity induced by radiotherapy or chemotherapy (Zhang et al., 2023). Mn^2+^ combined with anti-TGF-β/PD-L1 bispecific antibodies reduces drug resistance by enhancing both innate and adaptive immune pathways. Manganese ion-based “Metalloimmunotherapy” plays a very important role in antitumor treatment.

#### 2.3.3 Manganese ion-based antitumor nanodrugs

As a simple and effective immune stimulant, manganese and its derived nanomaterials have shown significant effects in tumor treatment. Various manganese-based nanomaterials have been widely used as nanocarriers to deliver immunotherapeutic agents, or as immunomodulators to reshape the immunosuppressive tumor microenvironment, or as immune activators to activate the body’s immune system directly or indirectly for clinical tumor immunotherapy. Compared with commonly used tumor immunotherapies such as tumor vaccines, immune checkpoint blockade therapy, adoptive cell therapy, manganese-based tumor immunotherapy has significant advantages in terms of stability, applicability, convenience, price, etc. In 2020, a research from Peking University showed that manganese ions could effectively synergistically enhance the effects of immune checkpoint inhibitors in various tumor models and significantly reduce the dosage of PD-1 antibodies (Lv et al., 2020). Translating NK cell therapies to treat solid tumors has proven challenging due to the tumor microenvironment (TME). Hypoxia in the TME induces immunosuppression that inhibits the cytotoxic function of NK cells. Thus, reversing hypoxia-induced immunosuppression is critical for effective adoptive NK cell immunotherapy. The particles (PLGA-MnO_2_ NPs) were developed by encapsulating MnO_2_ NPs into poly (lactic-co-glycolicacid) (PLGA), which can catalyze the degradation of endogenous H_2_O_2_ to produce oxygen to alleviate tumor hypoxia, resulting in significantly enhanced cytotoxicity of NK cells. This manganese ion-based NPs are promising new tools to improve adoptive NK cell therapy (Murphy et al., 2021). Manganese-based nanomaterials combined with immune activators can synergistically activate the cGAS-STING pathway to enhance antitumor immune effects while reducing the side effects caused by excessive manganese. Chen *et al.* reported a thio-cGAMP-Mn^2+^ nanovaccine, which enhanced the antitumor immune response through direct cytosolic co-delivery of cGAMP and Mn^2+^. The fixation of cGAMP with Mn^2+^ ions not only improve its stability, but also potentiate the activation of STING pathway. The nanovaccine increased the production of cytokines, and activated CD8^+^ T cell immunity, and in turn suppressed the primary and distal tumors growth through long-term immune memory and led to long-term survival of poorly immunogenic B16F10 melanoma mice (Chen et al., 2021).

### 2.4 The role of copper in antitumor therapy

#### 2.4.1 Physicochemical properties, physiological functions of copper and cuproptosis

Copper (chemical symbol: Cu) is a transition metal element with redox activity, with an atomic number of 29, and its main valences are Cu^+^ and Cu^2+^. Copper is a very important trace element in the human body, serving as a structural and catalytic cofactor for various enzymes, and is involved in regulating important life processes such as energy metabolism, antioxidation, neurotransmitter synthesis, and iron metabolism (Reznik et al., 2022; Schwarz et al., 2023). When Cu^+^ accumulates excessively in cells, it is oxidized by H_2_O_2_ to produce ·OH, which damages proteins, nucleic acids, and lipids within the cells, and interferes with the synthesis of iron-sulfur cluster proteins. Since iron-sulfur cluster proteins are essential for the activity of many important cellular enzymes, so excessive accumulation of copper in cells can be toxic. Therefore, maintaining copper homeostasis within cells is importantThe body’s homeostasis of copper must be precisely regulated by membrane transport systems (Campos et al., 2021). The concentration of copper within cells is strictly controlled by copper transport proteins and copper chaperones. Since only monovalent copper ions Cu^+^ are transportable, extracellular Cu^2+^ is often reduced to Cu^+^ before entering the cell. Copper transport proteins are responsible for the influx of Cu^+^ across the cell membrane, and copper transport enzymes α (ATP7A) and β (ATP7B) are necessary for expelling excess copper from the cell (Ash et al., 2021).

**FIGURE 5:**
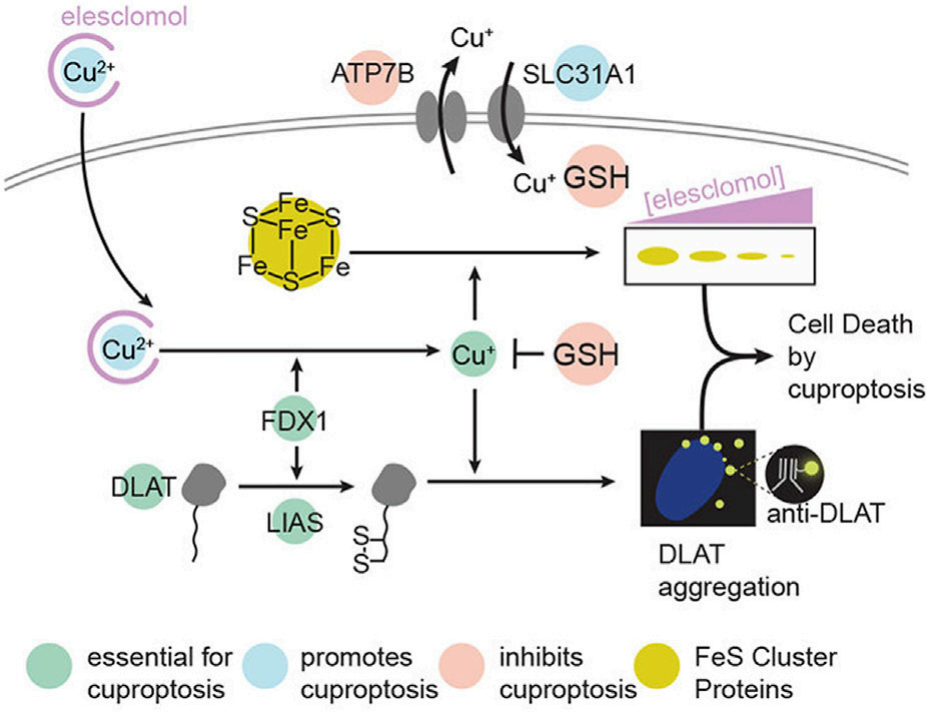
Schematic of mechanisms promoting cuproptosis. Reproduced with permission from ref (Tsvetkov et al., 2022), PDM 1.0. Copyright ^©^ 2022 by the authors.

Cuproptosis is a unique type of cell death published in Science recently. The mechanism of cuproptosis is different from other known forms of programmed cell death such as apoptosis, pyroptosis, necrosis, and ferroptosis. By binding to the tricarboxylic acid (TCA) cycle, intracellular copper accumulation triggers the aggregation of mitochondrial lipoylated proteins and the destabilization of Fe-S cluster proteins, which in turn causes a protein toxicity reaction leading to cell death (Tsvetkov et al., 2022) (as shown in Figure 5). Studies have shown that necessary factors for cuproptosis include the presence of glutathione, mitochondrial metabolism of galactose and pyruvate, and glutamine metabolism. Endogenous intracellular glutathione, as a thiol-containing copper chelator, can inhibit cuproptosis (Saporito-Magriñá et al., 2018).

#### 2.4.2 Antitumor mechanisms based on copper homeostasis imbalance

Studies have found that the content of copper in the serum and tissues of patients with various types of tumors is significantly higher than that in normal populations, and is related to tumor staging or progression (Ge et al., 2022). Appropriate high concentrations of copper ions promote tumor cell proliferation, angiogenesis, and metastasis. Both excessively high and excessively low concentrations of copper ions can promote cell death. Therefore, regulating the concentration of intracellular copper ions (copper deficiency and copper overload) has become an attractive new target for tumor treatment. Copper ions participate in the regulation of the structure and activity of copper-related proteins or enzymes, which are crucial for the survival and development of tumors. Copper deficiency can lead to impaired function of copper-binding enzymes and a lack of copper-related proteins, and all processes that lead to cellular copper depletion result in cellular dysfunction (Nyvltova et al., 2022). In addition, copper depletion can lead to energy and nutrient deficiency in cells, as well as increased oxidative stress and mitochondrial membrane rupture, all of which lead to tumor cell apoptosis. Given this, reducing the level of intracellular copper is a promising strategy for tumor treatment.

Furthermore, copper overload is another effective method for killing tumor cells. Copper overload can induce a Fenton-like reaction, mediating cell death. The Cu^+^-mediated Fenton-like reaction can proceed at a higher reaction rate over a wider pH range, with a reaction rate about 160 times faster than that of Fe^2+^ (Qiao et al., 2024). Copper overload can also mediate tumor cell death through the cuproptosis pathway. Cells dependent on glycolytic respiration are more sensitive to cuproptosis. With the consumption of glucose and glutathione, Cu^+^ binds to the dihydrolipoamide S-acetyltransferase (DLAT) protein more effectively, causing the formation of DLAT oligomers. The aggregation of DLAT oligomers can downregulate Fe-S cluster proteins, thereby leading to cuproptosis of tumor cells (Tsvetkov et al., 2022). Studies have shown that exogenous copper can disrupt redox homeostasis through changes in copper-dependent glutathione, enhancing ferroptosis (Du et al., 2022). In addition, copper ions can also regulate antitumor immune responses. The increase in Cu^2+^ not only promotes dendritic cell maturation but also enhances the antitumor effect mediated by cytotoxic CD8^+^ T cells (Zhao F. et al., 2023). This new mode of cell death, cuproptosis, also suggests that using copper ion metal carriers to inhibit mitochondrial respiration and kill tumor cells may become a new treatment method.

#### 2.4.3 Copper ion-based antitumor nanodrugs

**FIGURE 6:**
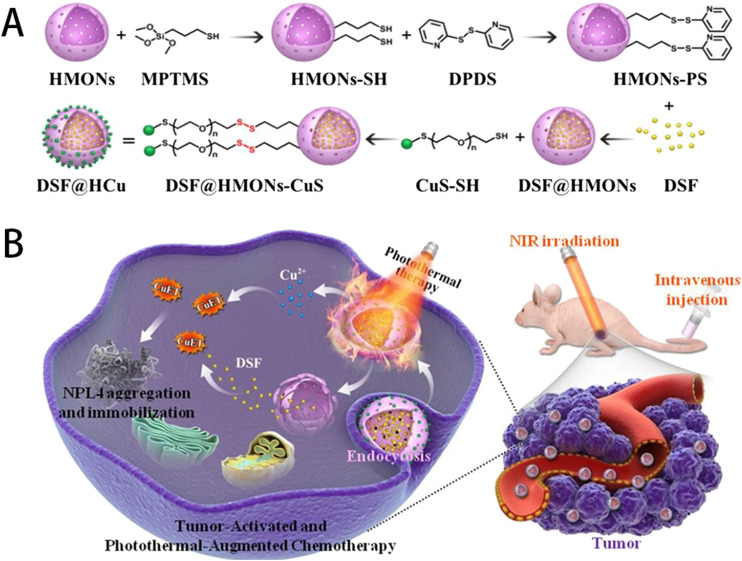
**(A)** Schematic illustration of the stepwise construction of DSF@HCu nanomedicine, and **(B)** NIR photothermal-augmented and in-situCu^2+^ release-activated DSF chemotherapy for the efficient killing of cancer cells and suppression of tumor growth with minimized side effects. Reproduced with permission from ref (Zhang et al., 2021), CC BY 4.0 Copyright ^©^ 2021 by the authors.

Copper-based compounds and nanomaterials possess excellent biocompatibility, can selectively target malignant tissues, and have strong permeability and high local retention rates. During the preparation process, their structure, composition, morphology, and size can easier to be controled and adjusted. These advantages make them widely used in tumor imaging and combined tumor therapy (Zhong et al., 2022). Zhang *et al.* utilized hollow mesoporous organosilica nanoparticles to integrate ultrasmall photothermal CuS particles onto the surface of the organosilica and the molecular drug Disulfiram (DSF) inside the mesopores and hollow interiors. The ultrasmall CuS acted as both photothermal agent under near-infrared (NIR) irradiation for photonic tumor hyperthermia and Cu^2+^ self-supplier in an acidic tumor microenvironment to activate the nontoxic DSF drug into a highly toxic diethyldithiocarbamate (DTC)-copper complex for enhanced DSF chemotherapy (as shown in Figure 6), which effectively achieved a remarkable synergistic *in-situ* anticancer outcome with minimal side effects (Zhang et al., 2021).

Chen *et al.* constructed CuS-pgh NMs by encapsulating copper sulfide nanoparticles using polylysine/glucose oxidase/hyaluronic acid shells (Chen et al., 2020), Liu *et al.* constructed a hybrid nanosystem composed of DNAzyme and Cu^2+^ (Liu et al., 2021), in which copper can mediate Fenton-like reactions to generate highly toxic hydroxyl radicals, enhancing thestarvation and chemodynamic tumor suppression effects. Cui *et al.* developed a safe, mitochondria-targeted copper-depleting nanoparticles. These nanoparticles can reduce the oxygen consumption and oxidative phosphorylation of triple-negative breast cancer (TNBC), promote glycolysis metabolism, reduce ATP production, cause mitochondrial membrane potential damage and increased oxidative stress, thereby induce the apoptosis of TNBC (Cui et al., 2021). A kind of nanochelator, Imi-OSi, were reported that can inhibit tumor angiogenesis through copper consumption, and form secondary particles through the phosphate/Cu^2+^ reaction polymerization to block blood vessels (Yang et al., 2019). For starvation-augmented cuproptosis and photodynamic synergistic therapy, a glucose oxidase (GOx)-engineered nonporous copper coordination nanomaterial GOx@[Cu(tz)] was developed. It inhibited 92.4% of tumor growth in athymic mice with bladder tumors, and the systemic toxicity produced was extremely low (Xu WJ. et al., 2022). Chang *et al.* designed a Ce6@AT-PEG-MSN-Pt (CAPMP) nanomotor, consisting of a janus platinum-mesoporous silica core, with acyl thiourea groups (copper chelators) conjugated with polyethylene glycol on the surface, and chlorin e6 (photosensitizer) in the pores, which can spontaneously move in tumor tissues, while performing the enhanced manipulation of the copper level, oxygen level, local temperature, and reactive oxygen species (ROS) level in the tumor microenvironment, to achieve effective tumor treatment (Chang et al., 2022). Nevertheless, copper-based nanomaterials also have their own limitations, and their instability and characteristics of easily oxidized under physiological conditions need to be solved, and the biosafetyneed to be improved (Tsymbal et al., 2022).

### 2.5 The role of zinc in antitumor therapy

#### 2.5.1 Physicochemical properties and physiological functions of zinc

Zinc (element symbol: Zn) is considered the second most abundant transition metal element in the body, after iron, with an atomic number of 30. Under physiological conditions, zinc exists in the form of divalent cations (Zn^2+^). Zinc is one of the essential trace elements required by the human body and is a core component of many proteins, playing the role of a “life gear” in the process of transporting substances and exchanging energy (Kambe et al., 2015). In mammalian cells, there are two forms of zinc: protein-bound zinc and free zinc. The former is used to maintain the catalytic activity and structural stability of metalloenzymes and transcription factors, while the latter acts as a signaling ion to regulate signal transduction (Maret, 2013). Therefore, zinc plays a significant role in many biological processes, including cell division, metabolic regulation, and immune responses. To maintain normal physiological functions, cellular zinc homeostasis is subtly coordinated by a set of zinc homeostasis regulatory proteins, including the Zrt-, Irt-like protein (ZIP) family for zinc influx, the zinc transporter (ZnT) family for zinc efflux, metallothioneins (MTs) for zinc intracellular storage, and metal-response-element-binding transcription factor (MTF)-1 for zinc cytosolic sensing (Colvin et al., 2010).

#### 2.5.2 Antitumor mechanism of zinc ion

It has been confirmed that the imbalance of zinc homeostasis is related to the development, invasion, and metastasis of various tumors (Bendellaa et al., 2024; Wang et al., 2020). Increasing evidence suggests that the elevation of intracellular zinc levels is mainly due to the overexpression of zinc transporters (ZIPs), which may promote the progression of certain specific cancers such as breast and pancreatic cancer. For example, ZIP6, ZIP7, and ZIP10 are highly expressed in breast cancer. Additionally, although zinc itself does not have redox activity, it can indirectly participate in the regulation of redox metabolism by acting as a catalytic or structural cofactor for proteins such as metallothioneins (MTs), copper/zinc superoxide dismutase (Cu/Zn-SOD), and the tumor suppressor protein p53. The balance of zinc homeostasis successfully activates antioxidant defenses, while zinc deficiency can disrupt protein structures and impair protein function, ultimately leading to oxidative stress and causing cell death. Accordingly, zinc chelation therapy mediated by zinc chelators can be used to treat certain types of cancer. Similar to zinc deficiency, zinc overload also has pro-oxidant properties. Excessive intracellular zinc can enter the mitochondria through the mitochondrial Ca^2+^ uniporter (MCU) (Medvedeva and Weiss, 2014), then irreversibly inhibit components of the electron transport chain (ETC.), which may stimulate the production of mitochondrial superoxide anions (·O_2_^−^) and damage mitochondrial function, leading to cell death. Furthermore, zinc overload can promote lysosomal dysfunction caused by increased lysosomal membrane permeability (LMP), which triggers cell death (Yu et al., 2009). Therefore, increasing intracellular zinc concentration is a viable therapeutic strategy to combat cancer.

#### 2.5.3 Zinc ion-based antitumor nanodrugs

Zinc ion-based nanodrugs for cancer treatment can deliver exogenous Zn^2+^ to tumor cells and induce cell death (Yao et al., 2023). PH-responsive zeolitic imidazolate framework-8 (ZIF-8), composed of 2-methylimidazole and Zn^2+^, can serve as a source of exogenous Zn^2+^ due to its excellent degradation ability (Gao L. et al., 2019). However, the hydrophobicity and relatively large size of ZIF-8 limit its antitumor application. Therefore, Lv *et al.* designed a multifunctional zinc-based nanoplatform, BSArGO@ZIF-8 NS, which can synergize ion interference and photothermal cancer treatment (Lv et al., 2022). BSArGO@ZIF-8 NSs can induce tumor cell apoptosis through intracellular Zn^2+^ overload, and at the same time, induce photothermal effects on cancer cells under (NIR) irradiation, showing higher lethality. In addition, Ding *et al.* synthesized ^F127^ZIF-8_CCCP_ nanoparticles for efficient cancer immunotherapy (Ding B. et al., 2023). ^F127^ZIF-8_CCCP_ nanoparticles achieve pH-sensitive Zn^2+^ release in tumor cells, leading to increased intracellular osmotic pressure and production of reactive oxygen species (ROS), which synergize with CCCP to strongly trigger caspase-1/GSDMD-dependent pyroptosis, thereby activating antitumor immunity and inhibiting tumor growth.

In addition to introducing Zn^2+^ exogenously into tumor cells, zinc overload caused by disrupting endogenous zinc homeostasis is another antitumor treatment strategy. Su *et al.* synthesized a series of platinum (IV) -terthiophene complexes for cancer chemotherapeutic immunotherapy (Su et al., 2023). Among these complexes, the cyclometalated platinum (IV) -terthiophene complex (Pt3) showed the best antiproliferative effect on triple-negative breast cancer (MDA-MB-231) cells. Mechanistically, Pt3 can induce DNA damage, disrupt intracellular zinc homeostasis, and disrupt intracellular redox homeostasis, ultimately activating tumor cell pyroptosis through the caspase-1/GSDMD pathway, and can activate antitumor immunity in mice (Riley and Tait, 2020).

In addition to inducing tumor cell death, zinc ions can also activate the cyclic GMP-AMP synthase (cGAS)-stimulator of interferon genes (STING) signaling pathway to enhance the effect of cancer immunotherapy. Therefore, various zinc-containing nanomaterials have been developed for zinc-based tumor immunotherapy. For example, Cen *et al.* prepared a pH-responsive nanocluster (ZnS@BSA) to enhance the immunotherapeutic effect against hepatocellular carcinoma (Cen et al., 2021). Zhang *et al.* reported a Zn^2+^-doped layered double metal hydroxide (Zn-LDH) to trigger a strong metal immunotherapy against solid tumors (Zhang et al., 2022). Ding *et al.* constructed a zinc-based metal-organic framework vaccine (ZPM@OVA-CpG) that controllably releases Zn^2+^, providing a promising strategy to improve the effect of immunotherapy (Ding B. et al., 2023). Zhao *et al.* prepared a pH-responsive and photoactive nanoparticle (ALA&Dz@ZIF-PEG) to enhance the effect of photodynamic immunotherapy (Zhao X. et al., 2023).

## 3 Metal-based antitumor nanoplatforms and multiionic combined interference therapy

Metal ion therapy, as an emerging tumor treatment method, has shown the potential to overcome the resistance of traditional chemotherapy drugs (Urbano-Gamez et al., 2024), In recent years, research on metal-ion-based nanomaterials and platforms has been emerging continuously (Table 1).

**TABLE 1:** Typical metal ions-based nanomaterial for tumor therapy in recent years.

Therapy strategy	Delivered metal ions	Nanomaterials	Size (nm)	Surface modification	Response	Applications of the metal ions	References
Ferroptosis	Fe^3+^	SPFeN	28 ± 4	PEG	808-nm NIR light	Generating ·OH by Fenton reaction, and achieving LPO	He et al. (2020a)
	Fe^2+^	PGFCaCO3-PEG	∼500	PEG	pH	The same as above	Han et al. (2022)
		Fe-ZnO_2_@HA (FZOH)	∼220	-	pH	Generating highly ·OH, inducing both ferroptosis and pyroptosis	Lu et al. (2024b)
	Fe^3+^ and Fe^2+^	Fe^2+^/Fe^2+^ loaded liposome (LPOgener)	94.6 ± 0.8	-	-	Generating ·OH by Fenton reaction, and achieving LPO	He et al. (2020b)
		Fe_3_O_4_-SAS@PLT	268.9 ± 8.9	Platelet membranes	-	The same as above	Jiang et al. (2020)
		Pt-FMO	∼90	-	pH	The same as above	Cheng et al. (2021)
		Ce6-PEG-HKN	∼410	PEG	laser irradiation	Synergistic effect of PDT and ferroptosis	Zhu et al. (2022)
		nano-activator (DAR) assembled by doxorubicin (DOX), tannic-acid (TA) and IR820	73.33 ± 4.95	-	laser irradiation	Promote ferroptosis and immunogenic cell death (ICD)	Xiong et al. (2021)
		USPBNPs@MCSNs (UPM)	∼200	-	pH	Generating ·OH by Fenton reaction, and achieving LPO	Zhao et al. (2024)
Calcium overload	Ca^2+^	CaO_2_@DOX@ZIF-67	mean diameter of 200	-	pH	Suppling O_2_ and H_2_O_2_, producing highly toxic ·OH through a Fenton-like reaction, resulting in improved chemodynamic therapy	Gao et al. (2019b)
		PEGCaCUR + US	∼140	PEG	pH, ultrasound	Binding to FDX1, inducing oligomerization of lipoylated DLAT, and mediating ROS-induced cell apoptosis as well	Zheng et al. (2021)
		M@CaCO_3_@KAE	∼100	Cancer cell membrane	-	Inducing mitochondrial damage, causing cytoskeleton collapse and oxidative stress, leading to apoptosis	Li et al. (2021)
		BSO-TCPP-Fe@CaCO3-PEG NPs	125.2 ± 7.7	PEG	pH, ultrasound	The same as above	Dong et al. (2020a)
		CaO_2_@ZIF-Fe/Ce6@PEG	∼110	PEG	H+, 660 nm light	The same as above	Shen et al. (2021)
		ZnPP@PAA-CaS NPs	122 ± 11	-	pH	Signaling transduction cascades to amplify the regulatory activity of chemical messengers and mediate antitumor immunotherapy	Zhao et al. (2021)
		CaO2@Cu-LA	80–100	CaO_2_	pH	Increasing ·OH radicals and NO molecules, disrupting the DNA structure and energy supply within tumor cells	Wang et al. (2024)
Immunotherapy	Mn^2+^	MnO@mSiO-iRGD NPS	132 ± 7	iRGD	pH	Mn^2+^-induced cGAS-STING pathway-activated immunotherapy	(Sun et al., 2022)
		amorphous porous Mn_2_P (APMP) NPs	∼180	Lipid layer	pH	The same as above	Hou et al. (2020)
		Mn-cGAMP nanovaccine	168 ± 20	-	-	The same as above	Chen et al. (2021)
		PLGA-MnO_2_NPs	116	PLGA	-	Reducing hypoxia and abundance of immunosuppressive metabolites, increasing NK cell activity	Murphy et al. (2021)
Cuproptosis	Cu^2+^	Perflourocarbon-/Ce6-loaded Cu@ZIF-8 (SonoCu)	∼100	Macrophage membrane	pH, ultrasound	Binding to FDX1, inducing oligomerization of lipoylated DLAT	Chen et al. (2023)
		Elesclomol/Cu coencapsulated polymer nanoparticles	62.8	An amphiphilic biodegradable polymer (PHPM)	ROS	The same as above	Guo et al. (2023)
		H-ferritin-Cu- regorafenib Nanoplatform	15.5	H-ferritin	pH	Inducing cuproptosis, resulting in a synergistical effect with regorafenib-mediated lethal autophagy	Jia et al. (2023)
		CuET-CuO@BSA	40.38 ± 0.17	BSA	ROS	Triggering ROS generation through Fenton-like reaction, inducing cuproptosis, enhancing the therapeutic effect of CuET	Wu et al. (2024a)
		ES@Cu(II)-MOF NPs	152.7	PEG	pH	Generating toxic ·OH and consuming endogenous GSH, triggering cuproptosis, triggering robust ICD, combing with an anti-PD-L1, achieving excellent antitumor effects	Lu et al. (2024a)
	Cu^+^	GOx@[Cu(tz)]	234	-	-	Binding to FDX1, inducing oligomerization of lipoylated DLAT	Xu et al. (2022a)
Zinc overload	Zn^2+^	BSArGO@ZIF-8 NSs	∼800	BSA	808-nm NIR light	increase of reactive oxygen species (ROS), initiating mitochondrial apoptotic, mediated ion-interference and photothermal combined therapy	Lv et al. (2022)
		^F127^ZIF-8_CCCP_	180	Pluronic F127	NIR irradiation	leading to increased intracellular osmotic pressure and production of reactive oxygen species (ROS)	Ding et al. (2023a)
		ZnS@BSA	≈100	BSA	-	activating cGAS/STING signals, leading to an improved immunotherapy efficacy	Cen et al. (2021)
		Zn-LDH	10	-	-	triggering a strong metal immunotherapy against solid tumors	Zhang et al. (2022)
		ZPM@OVA-CpG	148	-	pH	stimulating cGAS-STING, improving the effect of immunotherapy	Ding et al. (2023b)
		ALA&Dz@ZIF-PEG	∼60	-	-	leading to the damage, releasing of mtDNA and activating the innate immune response	Zhao et al. (2023b)
Chemodynamic therapy	Fe2^+^	DOX/GA-Fe@CaCO_3_-PEG	∼109.2	PEG	pH	Achieving CDT and subsequently reducing DOX efflux by retarding ATP production	Dong et al. (2020b)
	Cu^2+^	MoS_2_-CuO@BSA heteronanocomposites	∼130	BSA	808-nm NIR light	Generating ·OH by Fenton reaction	Jiang et al. (2021)
	Cu^+^	Cu_2_O@CaCO_3_NCs	∼100	HA	pH, H2S, 1,064-nm NIR-II light	The same as above	Chang et al. (2020)
	Mn^2+^	mPEG-b-PHEP@ZnxMn1-xS (PPIR780-ZMS)	∼150	mPEG-b-PHEP	808-nm light	The same as above	Li et al. (2022)
	Cu^2+^	Hollow mesoporous organosilica NPs DSF@HCu	98.4	Organosilica	near-infrared (NIR) irradiation	Combination of photonic tumor hyperthermia with *in-situ* Cu^2+^-activated highly toxic diethyldithiocarbamate (DTC)	Zhang et al. (2021)
	Fe^3+^ and Fe^2+^	Semiconducting polymer nanocomposites (SPFeN_OC_)	42.1	Surface camouflaging of Hybrid cell membranes of cancer cells and osteoclasts	Ultrasound Irradiation	Mediating CDT by producing ·OH in tumor microenvironment and Enabling SDT by generating ^1^O_2_	Zhang et al. (2024)
Pyroptosis	Fe^3+^	DOPC-coated MIL-100(Fe)	∼250	-	pH	Inducing cell pyroptosis, Potential immunotherapy	Ploetz et al. (2020)
synergistic PDT/CT/CDT therapy	Ca^2+^	CaO_2_@DOX@ZIF@ASQ	625	CaO_2_	pH	Generating both ROS and O_2_,attenuating the hypoxic condition and potentiates PDT/CT/CDT synergistic cancer therapy	Yu et al. (2024)

Metal nanomaterials are forming a drug-free nanoplatform, in which metal ions are gradually replacing traditional drugs as part of the construction of nanomaterials (Chuang et al., 2023; Lei et al., 2023; Li et al., 2024; Naletova et al., 2023). These nanoparticles can specifically deliver metal ions to tumor cells, thereby reducing damage to normal tissues, improving treatment effects, and reducing side effects. In addition, nanoparticles can also enhance the solubility, stability, and biocompatibility of metal ions through their surface modification and functionalization, further optimizing the application of metal ion therapy. Additionally, the nanoplatform is capable of delivering multiple components simultaneously, achieving synergistic effects of different therapeutic mechanisms. Metal ions in combination with other treatment strategies can be used to design new synergistic anti-tumor regimens, improving tumor treatment outcomes, and reducing the potential toxicity of drugs to normal tissues. Additionally, strategies that modulate the homeostasis of multiple metals have shown promising anti-tumor effects. Recently, there has been considerable research on regulating the homeostasis of various metals for cancer treatment. For instance, Shen *et al.* reported a pH-responsive nanoplatform (CaO_2_@ZIF-Fe/Ce6@PEG) that simultaneously causes overload of iron and calcium within tumor cells, providing a robust self-supplied ROS pathway to further enhance the efficacy of CDT/PDT (Shen et al., 2021). Xu *et al.* constructed a novel copper/iron mixed hollow nanoplatform (DOX@Fe/CuTHHaMOF) to disrupt intracellular copper/iron metabolism and amplify intracellular oxidative stress, which effectively suppresses tumor growth through synergistic copper apoptosis/iron apoptosis/apoptosis (Xu Y. et al., 2022). Zheng *et al.* developed a Ca^2+^/Mn^2+^ ion reservoir (PEGCaMnUA) to increase intracellular oxidative stress, enhancing the effect of ion interference therapy (IIT) (Zheng et al., 2023). Deng *et al.* prepared a Ca&Mn dual-ion mixed nanostimulator (CMS) to simultaneously activate iron apoptosis and innate immunity, providing a new strategy for effective immunotherapy against triple-negative breast cancer (TNBC) (Deng et al., 2024). Huang *et al.* constructed a Mn/Zn dual-metal nanoplatform (PMZH) to activate the cGAS-STING signaling pathway and ROS-mediated tumor cell death, achieving enhanced tumor immunotherapy (Huang Y. et al., 2023). Wang *et al.* prepared a carrier-free nanodrug (Mn-ZnO_2_ nanoparticles) for delivering zinc-manganese dual ions for the treatment of p53-mutated tumors (Wang J. et al., 2023). In summary, these strategies based on disrupting the homeostasis of multiple metals within cells will provide innovation for cancer treatment. These strategies are expected to address the issue of multiple drug resistance in tumors. In conclusion, combining the advantages of nanotechnology, metal ions have emerged as a new strategy for anticancer therapy.

## 4 Advantages of metal-based nanomaterials

Metal-based nanomaterials have been proven to have tremendous potential as targeted drug delivery systems, imaging agents, and therapeutics. Due to their unique advantages, metal nanomaterials have broad application prospects and significant research value in numerous fields. Compared to other types of nanomaterials, metal-based antitumor nanomaterials have the following advantages. First and foremost, metal-based nanomaterials can release a large amount of specific metal ions in the tumor microenvironment, thereby disrupting the metal ion balance required for cellular function. Therefore, metal-based nanomaterials hold great promise for enhancing the imbalance of metal homeostasis in cancer cells. Currently, metal-based nanomaterials under study either through metal chelation or metal overload lead to the disruption of metal homeostasis in cancer cells, ultimately resulting in cell death through various means such as apoptosis, pyroptosis, ferroptosis, cuproptosis, et al. Secondly, due to their small size and high surface area-to-volume ratio, metal nanomaterials exhibit unique physical, chemical, and optical properties, such as strong near-infrared absorption, magnetothermal effects, and the ability to easily accumulate, separate, or undergo targeted movement and localization. These properties enable metal nanomaterials to play a unique role in tumor diagnosis and treatment. For example, gold nanomaterials can rapidly heat up under laser irradiation through the photothermal conversion effect, thereby killing tumor cells and achieving photothermal therapy (Khoobchandani et al., 2020). Magnetic nanomaterials, on the other hand, can indirectly kill tumor cells through the magnetothermal effect, showing good antitumor effects. Furthermore, metal nanomaterials have a high surface area and abundant active sites, which can serve as carriers for anticancer drugs, enhancing the uptake of drugs by cancer cells and improving the efficacy of cancer treatment while reducing the dosage of antitumor drugs. Surface modification of metal nanomaterials can also further enhance their biocompatibility and safety, reducing their potential systemic side effects. Additionally, metal nanomaterials have shown great potential in tumor immunotherapy. For instance, by preparing ultra-small metal-organic nanomaterials, it is possible to achieve sufficient accumulation at the tumor site and rapid renal clearance, enhancing treatment efficacy and greatly reducing long-term toxicity caused by body retention. At the same time, by concentrating multiple functional drug molecules on a single metal nanoplatform, the preparation of multifunctional integrated nanohybrid materials is expected to achieve multimodal synergistic tumor treatment, improving cancer treatment efficiency and imaging resolution. In summary, metal-based antitumor nanomaterials, due to their unique physicochemical properties, high surface area-to-volume ratio, and abundant active sites, as well as their multifunctionality, tunability, and ability to interact with cellular processes, make them attractive candidates for targeted cancer therapy. They have a unique role in tumor diagnosis and treatment and show a broad application prospect.

## 5 Clinical trials of metal nanomaterials for tumor treatment

Magnetic iron oxide nanoparticles are the earliest metal nanoscale agents applied clinically. They were initially used to treat clinical iron deficiency anemia, with a clinical history dating back to 1930, accumulating valuable data on safety and side effects. In the early 21st century, there was evidence that systemic exposure to iron oxide nanoparticles could induce anti-cancer immune effects. By directly injecting biocompatible iron oxide nanoparticles into tumors and then stimulating them with alternating magnetic fields to generate heat, i.e., using magnetic nanoparticles (Nano-Cancer^®^ therapy) for intratumoral hyperthermia, tumor growth could be suppressed. Given this mechanism, this method was used clinically in the treatment of glioblastoma and prostate cancer about 20 years ago (Johannsen et al., 2007a; Johannsen et al., 2007b; Maier-Hauff et al., 2007). Subsequently, researches on magnetic iron oxide nanoparticles in tumor treatment has become more in-depth, with new formulations continuously emerging and complex mechanisms of action being revealed (Savitsky and Yu, 2019; Soetaert et al., 2020). For example, Nanotherm™ (EU) is used for the clinical treatment of recurrent neuroblastoma (Maier-Hauff et al., 2011). Zanganeh et al. revealed the hidden intrinsic therapeutic effects of iron oxide nanoparticle compounds ferumoxytol (an FDA-approved drug) on tumors (Zanganeh et al., 2016). After mixing ferumoxytol with tumor cells and co-injecting them into mice, compared to tumor cells not injected with ferumoxytol, the growth rate of the tumor was significantly slowed down. Furthermore, they demonstrated that systemic exposure to ferumoxytol in T cell-deficient mice, followed by intravenous injection of small cell lung cancer (SCLC) cells, could prevent liver metastasis. They concluded that the intrinsic therapeutic effect of ferumoxytol on cancer growth comes from the polarization of macrophages to a pro-inflammatory M1 phenotype. In other words, they showed that the innate immune cells in the experimental model tumor microenvironment respond to iron oxide nanoparticles, responsible for the anti-tumor immune effect, rather than T cells. In summary, iron oxide nanoparticles are truly effective agents for hyperthermia and immunotherapy for cancer.

Additionally, there are reports of clinical trials using gold nanoparticles as precious metals for breast cancer patients. The Nano Swarna Bhasma (NSB) Nano-Ayurvedic medicine-gold nanoparticles-based drug was developed using proprietary combinations of gold nanoparticles and phytochemicals through innovative green nanotechnology for human metastatic breast cancer patients. Patients treated with the NSB drug capsules along with the “standard of care treatment” (group B) exhibited 100% clinical benefits when compared to patients in the treatment group A, thus indicating the tremendous clinical benefits of NSB drug in adjuvant therapy (Khoobchandani et al., 2020). The results also indicate that Nano Swarna Bhasma can be safely used as a valuable adjuvant therapeutic agent to reduce the adverse effects of routine chemotherapeutic agents while providing measurable therapeutic efficacy in treating breast and other forms of human cancers.

## 6 Limitations and challenges in clinical translation of metal nanomaterials for tumor treatment

Although metal nanomaterials show great potential for tumor treatment at present, they still have many limitations. Firstly, although many anti-tumor mechanisms of metal ion based nanodrugs have been reported, but most of them have only been validated in cell lines. Therefore, further research on these mechanisms in more organoid and animal models is necessary to deeply elucidate the anti-tumor mechanisms and effects of metal ion-based nanodrugs. However, the lack of animal models that can accurately simulate human tumor conditions is one of the recognized deficiencies in the field, leading to a weak correlation between preclinical studies and clinical trial results. For this reason, researchers are trying to establish organoids from patients, which are innovative screening devices as close as possible to the *in vivo* environment. The established platform takes into account tumor angiogenesis and the 3D microenvironment, so as to select the best anti-tumor therapy for patients through the screening device, and collect lymphocytes from the patient’s blood to test the effectiveness of immunotherapy. Secondly, there are a series of biological barriers in the body, and if nanocarriers cannot efficiently pass through them, they will also limit the therapeutic effect; interactions between nanomaterials and biological entities in the blood can alter the physicochemical characteristics and stability of nanodrugs, hindering the specific binding of targeting molecules to receptors; most nanoparticles are taken up and cleared by macrophages in the liver or spleen after entering the body, preventing their further delivery to tumor tissues. Therefore, this also poses higher requirements for the design and development of clinical drugs. Lastly, the biological safety of nanomedicines *in vivo* remains the greatest challenge for their clinical application. Currently, most excellent studies have only examined the short-term toxicity of nanomedicines and the corresponding structural damage to major organs, lacking experiments on the long-term toxicity of metal nanomedicines. Therefore, it is necessary to conduct comprehensive studies on their absorption, biodistribution, metabolism, excretion, clearance, long-term tissue accumulation, chronic toxicity, and dose-dependent toxicity before clinical translation. In fact, metal nanomaterials for multimodal combination therapy have shown advantages of synergistic enhanced therapeutic effects and low cytotoxicity. By selecting metal ions with high biocompatibility (e.g., Ca^2+^, Fe^2+/3+^, Zn^2+^, etc.) and endogenous bioactive molecules as ligands, toxicity can be effectively prevented (Lai et al., 2021; Simon-Yarza et al., 2018). The additional cargo that may be loaded also needs to be considered, as they may pose a threat to the organism. In addition, there is an increasing need for extensive and in-depth research into the degradation mechanisms and pathways of metal nanomaterials *in vivo*.

Additionally, the synergistic and antagonistic effects among carriers used in cancer treatment, the metal nanoparticles embedded within them, and the complexes containing these metal ions remain unknown. Knowledge of the chemical interactions between metal nanoparticles and these metal ion complexes is still limited. There is currently no answer to the question of whether it is possible to precisely control the release of metal ions from the nanoparticle surface while increasing the biocompatibility of the materials and maximizing their anticancer activity. The properties of organic-inorganic systems, ligand substitution on the surface of metal nanoparticles, and the behavior of maintaining complex stability in the presence of biofluids also remain unexplored.

The clinical translation of metal ion-based nanodrugs for tumor treatment still faces some challenges. Although the valuable experience of intravenous injection of iron oxide nanoparticles for the treatment of anemia is a guide, clinical product iterations have also been carried out in subsequent studies to gain relevant knowledge and sufficient experience. However, nearly a century and the rich clinical experience gained from “three generations” of successful iron oxide nanoparticle formulations have not led to substantial progress in the field of metal nanoparticle anti-tumor drugs as expected. Even with such a long clinical history, new discoveries of complex biological interactions with iron oxide nanoparticles still indicate that our understanding of the complexity of cancer is insufficient. The interactions between nanoparticles and biological systems remain unknown, and incorporating these interactions into the design and function of cancer nanomedicine has greater potential than focusing solely on their engineering design. Cancer is a family of complex diseases, with considerable heterogeneity between subsets, within subsets of the same disease, and within individual patients (Tomasetti and Vogelstein, 2015). The tumor microenvironment is heterogeneous, dynamic, and complex, so each nanoparticle formulation must be evaluated in the context of a wide range of biological scenarios. In addition, preclinical data is not a universally reliable indicator of clinical benefit. Data suggest that more caution is needed when selecting preclinical animal models and that preclinical results need to be carefully assessed.

The historical reality of cancer treatment research and development is that most product concepts fail to translate into clinical applications. The development of small molecule cancer therapeutics has not yet overcome the challenges posed by tumor metastasis, which increases the need for rigorous clinical validation of preclinical data. Metal nanoparticles have great potential for anti-tumor effects, but they must also be modified and designed under the premise of a clear understanding of the specific disease context and interaction with host immune biology, and require the adoption of different research perspectives and multidisciplinary collaboration to achieve efficient therapeutic outcomes.

## 7 Conclusion and perspectives

In this review, we summarize the mechanisms of metal ions (Fe^2+/3+^, Ca^2+^, Mn^2+^, Cu^2+^ and Zn^2+^) in anti-tumor therapy, and the nanodrugs developed based on these ions over the past 5 years. We analyze the challenges faced by the clinical translation of current metal-based anti-tumor nanomaterials and their future application prospects, and propose suggestions to address related defects. With the proposal and elucidation of concepts such as ferroptosis and cuproptosis, research on metal ion-based anti-tumor nanomaterials has emerged, becoming a new research hotspot. Metal-based anti-tumor drugs can not only cause cell death individually by inducing intracellular ion imbalances but can also enhance the effects of other anti-tumor therapies such as chemotherapy, radiotherapy, photothermal therapy, and photodynamic therapy. They play a role in comprehensive tumor treatment and achieve synergistic effects of different treatment mechanisms, and demonstrate significant potential. However, many obstacles need to be addressed in various aspects such as the selection of model drugs, the development of preclinical research models, drug safety *in vivo*, and patient screening in clinical research *et al.* The continuous development of new nanomaterial manufacturing processes and standardized procedures, as well as new experimental technologies such as organoid culture systems, will further promote the development of metal nanodrugs in the field of anti-tumor therapy.

With in-depth research on the mechanisms of cancer pathogenesis and the action of metal-based drugs, the design of metal nanodrugs will become more precise and efficient. The application of new strategies and technologies will further promote the development of metal nanodrugs in the field of anti-tumor therapy, and the application of metal nanodrugs in the field of anti-tumor therapy will become more extensive and profound.
